# Exploration of Adhesion Molecule Expression in Cardiac Muscle of Early Atherosclerosis Dyslipidemic Sprague Dawley Rats

**DOI:** 10.2174/1874104501812010124

**Published:** 2018-10-24

**Authors:** Titin A. Wihastuti, Fitria N. Aini, Nurul C. Lutfiana, Teuku Heriansyah, Nafisatuz Zamrudah

**Affiliations:** 1 Departement of Biomedicine, Faculty of Medicine, Brawijaya University, Malang, Indonesia; 2 Departement of Cardiology, Faculty of Medicine, Syiah Kuala University, Aceh, Indonesia

**Keywords:** ICAM-1, VCAM-1, Dyslipidemia, Molecule expression, Adhesion, Atherosclerosis, Immunofluorescence

## Abstract

**Objective:**

This study is aimed to examine the expression of ICAM-1 and VCAM-1 in cardiac tissue of dyslipidemic Sprague Dawley rats.

**Methods:**

Eight Sprague Dawley strain rats, with 150-200 gram body weight, were divided into two groups. The control group was fed a standard diet, the positive control group was fed a high-fat diet as our previous study for 8 weeks. The pattern of distribution of ICAM-1 and VCAM-1 in cardiac muscle cell was examined by immunofluorescence and observed with a confocal laser scanning microscope. Lipid profile was also examined at the end of the study.

**Result:**

Independent t-test showed no differences in ICAM-1 and VCAM-1 expression in cardiac muscle of hypercholesterol-diet-fed Sprague Dawley rat compared to control.

**Conclusion:**

The expression of ICAM-1 and VCAM-1 in cardiac muscle did not change after the onset of atherosclerosis.

## 1 INTRODUCTION

Ischemic heart disease and stroke recently have been known as the leading cause of death in the world (World Health Organization, 2014) [1-3].Stroke occurs in 1 out of 20 causes of death in US every year (American Heart Association, 2015). Additionally, stroke also becomes the most leading causes for long-term disability [3]. Cardiovascular disease and stroke are caused by several risk factors such as smoking, physical inactivity,obesity, hypertension, diabetes, and dyslipidemia [4].

Dyslipidemia is established as a major risk factor for CVD and stroke [5]. Dyslipidemia is a lipid metabolic impairment condition caused by interaction between genetic and environmental factors, which increases accumulation of Low-Density Lipoprotein (LDL) in the blood. In endothelial cells, LDL undergoes modification and becomes Ox-LDL, as a result of macrophage and endothelial cells activities. A lipid that accumulates in the endothelium becomes the basis for endothelial dysfunction leading to the formation of atherosclerosis [6].

Recently one of emerging theories beyond the formation of atherosclerosis that is widely studied is the involvement of lipoprotein-associated phospholipase A2 (Lp-PLA2) [7-9]. Lp-PLA2 is divided into secreted Lp-PLA2 and found in the circulating system and latent Lp-PLA2 located within atherosclerotic plaque. Approximately 70% of secreted Lp-PLA2 binds to LDL-C and hydrolize LDL into lysophosphatidylcholine (Lyso-PC). While Lp-PLA2 in atherosclerotic plaque hydrolize oxidized LDL (oxLDL) into Lyso-PC and oxidized non-Esterified fatty acid (Ox-NEFA)^9^. Lyso-PC and Ox-NEFA then stimulate the expression of adhesion molecules, upregulate inflammatory cytokines, enhance matrix metalloproteinase expression, amplify oxidation and expand necrotic lipid core and thinning fibrous cap [10].

Intracellular cell adhesion molecule-1 (ICAM-1) and Vascular cell adhesion molecule-1 (VCAM-1) are immunoglobulin superfamily members that play important roles in adhesion of leukocytes to vascular endothelium [11]. ICAM-1 has been known to be upregulated at the onset of pressure overload by aortic constriction and in the presence of cardiac inflammation mediates T-cell recruitment during pathologic cardiac remodelling in heart failure [12-14]. VCAM-1 is strongly upregulated in the heart during acute and chronic stages of diseases such as in inflammatory cardiomyopathy [15]. This study was aimed to explore ICAM-1 and VCAM-1 expression of cardiac tissue from the early progression of atherosclerosis in dyslipidemia in Sprague Dawley rat model.

## 2 MATERIALS AND METHODS

### 2.1 Study Design

Eight Sprague Dawley strain rats, 6-8 weeks old, with 150-200 gram body weight, were obtained from Bogor Agricultural University, Indonesia. Rats were divided into two groups after acclimatization. The control group was fed a standard diet (N), the positive control group was fed a High-Fat Diet (HFD) as our previous study [16] (DL) for 8 weeks. This study was conducted at the Biomedical Laboratory and Central Laboratory of Biological Sciences, Brawijaya University after obtaining ethical clearance assessment by the Health Research Ethics Committee.

### 2.2 ICAM-1, and VCAM-1 Measurement

Heart organ was separated from the pulmonary artery, pulmonary vein, aorta, and vena cava fixed with PHEMO buffer (68 mM PIPES, 25 mM, HEPES, pH 6.9, 15 mM EGTA, 3 mM MgCl2, 10% [v/v] dimethyl sulfoxide containing 3.7% formaldehyde and 0.05% glutaraldehyde). Each parameter was then processed for imumunofluoresence with VCAM-1 anti-rat antibody using fluorescein isothiocyanate secondary antibody and ICAM-1 anti-rat antibody using rhodamin secondary antibody (BIOS Inc., Boston, MA, USA). The luminescences were observed with confocal laser scanning microscopy (Olympus Corporation, Tokyo, Japan) and were quantitatively analyzed by Olympus FluoView software (version 1.7A; Olympus Corporation) Fig. (**1**).

### 2.3 Statistical Analysis

This study used independent T-test to analyse VCAM-1, and ICAM-1 expression in cardiac muscle of Sprague Dawley strain *R. norvegicus* rats with hypercholesterol administration using SPSS software (v 20; IBM Corporation, Armonk, NY, USA).

## 3 RESULT AND DISCUSSION

**Fig. (2):**
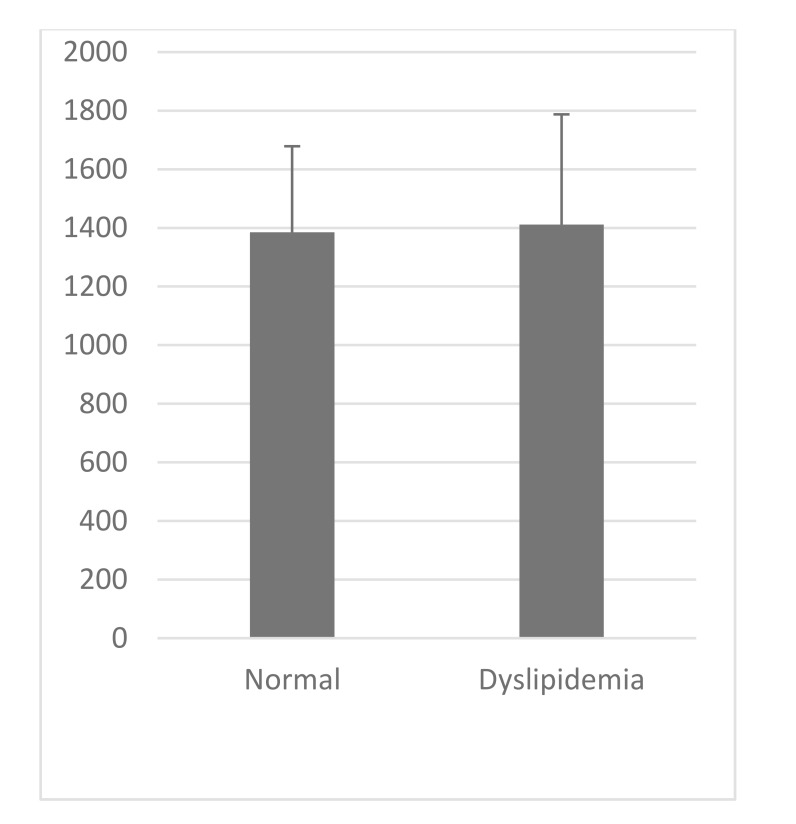
ICAM- 1 expression in cardiac muscle.

**Fig. (3):**
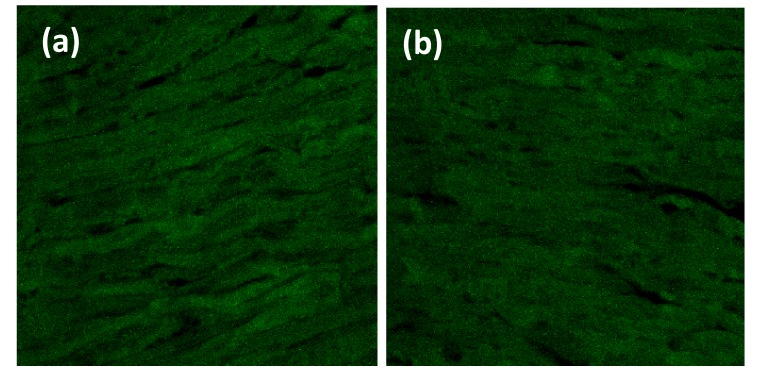
Qualitative observation of VCAM-1 expression in cardiac muscle cell (a) dyslipidemia rat (b) control rat.

VCAM-1 expression in the normal group was similar to that of high-fat diet group (Table **1**, Figs. **2** and **3**). Likewise, the expression of ICAM-1 in the normal group was not significantly different compared to the high-fat diet group.

High fat diet induces abnormal amount of lipids in the blood or dyslipidemia causing hyperlipidemia which is characterized by elevation of total cholesterol and/or elevation of low-density lipoprotein (LDL) cholesterol and/or elevation of triglyceride concentrations and/or decrease of high-density lipoprotein (HDL) cholesterol in the blood [17]. Lipid profile result from our previous publication was in line with this theory [18]. Lipid is normally stored in adipose tissue as an esterified lipid, but in high-fat diet, 40%-50% of lipid undergoes spillover causing ectopic fat deposition [23]. Fat deposition in blood vessels causes infiltration of apoB containing lipoproteins (LDL, remnant VLDL, and chylomicrone) in the artery wall. The retained lipoproteins induce ROS production that lead to endothelial dysfunction. ROS then modify LDL into oxLDL and oxLDL which then induce more ROS formation [19].

Oxidative stress has an important role in the pathophysiology of atherosclerosis. Sufficient levels of ROS, particularly superoxide anion and hydrogen peroxide have been shown to activate nuclear factor kappa beta (NF-κB) [20]. NF-kB are transcription factors which bind to VCAM-1 and ICAM-1 promoter and induce VCAM-1 and ICAM-1 gene transcription. This process promotes the binding, rolling and stable arrest of inflammatory white blood cells, such as T cells, monocytes and mast cells [21]. Cytokine production by inflammatory cells within endothelial like TNF-α and IL-1 also works on endothelial cell and induces endothelial cells to express VCAM-1 and ICAM-1 via NF-κB activation. ICAM-1 and VCAM-1 play a major role in the initiation of early atherosclerosis, mainly its contribution to monocyte adhesion. Inhibition of the inflammatory response is widely known to be beneficial in the early stages of atherosclerosis [22].

## CONCLUSION

Results from our study show that there were no differences between ICAM-1 expression in dyslipidemia group compared to the normal group and no differences were observed between VCAM-1 expression in dyslipidemia group compared to the normal group. ICAM-1 and VCAM-1 in aorta may have an important role in the initiation of atherogenesis, while in cardiac tissue, ICAM-1 and VCAM-1 are reported to be upregulated in several studies at the onset of pressure overload by aortic constriction and in the presence of cardiac inflammation such as in myocardial infarction or inflammatory cardiomyopathy [12-15]. Soluble VCAM-1 also is an established biomarker to predict future or long-term acute coronary syndrome [23-25]. While the cardiac effect of atherosclerosis is not directly due to atherosclerosis in itself, the cardiac effect of atherosclerosis is due to the inflammatory response of an infarct due to aortic constriction or coronary artery constriction. Therefore, adhesion molecule expression ICAM-1 and VCAM-1 was not upregulated in the early progression of atherosclerosis and they are less likely to contribute to cardiomyopathy because of dyslipidemia.

## Figures and Tables

**Fig. (1):**
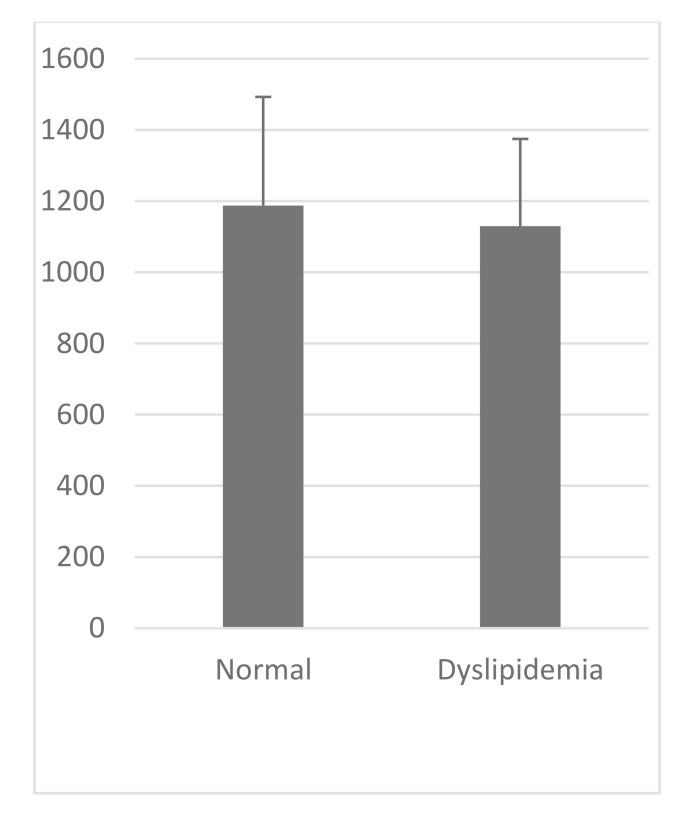
VCAM-1 expression in cardiac muscle.

**Table 1:** Parameter measurement, Independent T-test for each group.

**Parameter**	**VCAM-1 (au)**	**ICAM-1 (au)**	
**Normal 8 Week** **(Mean±SD)**	1187.49±305,33	1384.89±293,59	
**Dyslipidemia 8 Week** **(Mean±SD)**	1129,47±245,539	1411,05±375,94	
**P Value**	>0.05	>0.05	

Data are presented as mean ± standard deviation (range) values. ICAM-1 = . Intracellular cell adhesion molecule-1; VCAM-1= . Vascular cell adhesion molecule-1; aU: arbritrary Unit

## References

[1] WHO (2014). Global status report on noncomunicable disease.

[2] WHO (2015). Cardiovascular Diseases. Geneva.

[3] American Heart Association Statistics Committee and Stroke Statistics Subcommittee (2016). Heart disease and stroke statistics—2016 update: A report from the American Heart Association.. Circulation.

[4] American Heart Association Statistics Committee and Stroke Statistics Subcommittee (2014). Heart Disease and Stroke Statistics—2014 Update: a report from the American Heart Association.. Circulation.

[5] Lam J.Y.T. (2012). Atherosclerosis.

[6] Cai A., Zheng D., Qiu R., Mai W., Zhou Y. (2013). Lipoprotein-associated phospholipase A2 (Lp-PLA(2)): A novel and promising biomarker for cardiovascular risks assessment.. Dis. Markers.

[7] Tousoulis D., Papageorgiou N., Androulakis E., Stefanadis C. (2013). Lp-PLA2--a novel marker of atherosclerosis: To treat or not to treat?. Int. J. Cardiol..

[8] Ikonomidis I., Kadoglou N.N., Tritakis V., Paraskevaidis I., Dimas K., Trivilou P., Papadakis I., Tzortzis S., Triantafyllidi H., Parissis J., Anastasiou-Nana M., Lekakis J. (2014). Association of Lp-PLA2 with digital reactive hyperemia, coronary flow reserve, carotid atherosclerosis and arterial stiffness in coronary artery disease.. Atherosclerosis.

[9] Samanta U., Bahnson B.J. (2008). Crystal structure of human plasma platelet-activating factor acetylhydrolase: Structural implication to lipoprotein binding and catalysis.. J. Biol. Chem..

[10] Suckling K. (2010). Phospholipase A2s: Developing drug targets for atherosclerosis.. Atherosclerosis.

[11] Hansson G.K. (2001). Immune mechanisms in atherosclerosis.. Arterioscler. Thromb. Vasc. Biol..

[12] Gerhardt T., Ley K. (2015). Monocyte trafficking across the vessel wall.. Cardiovasc. Res..

[13] Niessen H.W., Lagrand W.K., Visser C.A., Meijer C.J., Hack C.E. (1999). Upregulation of ICAM-1 on cardiomyocytes in jeopardized human myocardium during infarction.. Cardiovasc. Res..

[14] Kuwahara F., Kai H., Tokuda K., Niiyama H., Tahara N., Kusaba K., Takemiya K., Jalalidin A., Koga M., Nagata T., Shibata R., Imaizumi T. (2003). Roles of intercellular adhesion molecule-1 in hypertensive cardiac remodeling.. Hypertension.

[15] Grabmaier U., Kania G., Kreiner J., Grabmeier J., Uhl A., Huber B.C., Lackermair K., Herbach N., Todica A., Eriksson U., Weckbach L.T., Brunner S. Soluble vascular cell adhesion molecule-1 (VCAM-1) as a biomarker in the mouse model of experimental autoimmune myocarditis (EAM).. PLoS One.

[16] Heriansyah T., Wihastuti T., Anita K., Iskandar A., Suhendra R., Setiabudi P. (2015). Atherogenesis inhibition by darapladib administration in dyslipidemia model Sprague-Dawley rats.. Natl. J. Physiol. Pharm. Pharmacol..

[17] Andri Wihastuti T., Sargowo D., Heriansyah T., Eka Aziza Y., Puspitarini D., Nur Iwana A., Astrida Evitasari L. (2015). The reduction of aorta histopathological images through inhibition of reactive oxygen species formation in hypercholesterolemia rattus norvegicus treated with polysaccharide peptide of *Ganoderma lucidum.*. Iran. J. Basic Med. Sci..

[18] Heriansyah T., Adam A., Wihastuti T., Rohman M. (2017). Elaborate evaluation of serum and tissue oxidized LDL level with darapladib therapy: A feasible diagnostic marker for early atherogenesis.. Asian Pac. J. Trop. Biomed..

[19] Thompson A., Gao P., Orfei L., Watson S., Di Angelantonio E., Kaptoge S., Ballantyne C., Cannon C.P., Criqui M., Cushman M., Hofman A., Packard C., Thompson S.G., Collins R., Danesh J., Lp-PLA(2) Studies Collaboration (2010). Lipoprotein-associated phospholipase A(2) and risk of coronary disease, stroke, and mortality: Collaborative analysis of 32 prospective studies.. Lancet.

[20] Kleber M.E., Siekmeier R., Delgado G., Grammer T.B., Winkelmann B.R., Scharnagl H., Boehm B.O., März W. (2015). C-reactive protein and lipoprotein-associated phospholipase A2 in smokers and nonsmokers of the Ludwigshafen Risk and Cardiovascular Health study.. Adv. Exp. Med. Biol..

[21] Hassan M. (2015). STABILITY and SOLID-TIMI 52: Lipoprotein associated phospholipase A2 (Lp-PLA2) as a biomarker or risk factor for cardiovascular diseases.. Glob. Cardiol. Sci. Pract..

[22] Kim J.A., Montagnani M., Chandrasekran S., Quon M.J. (2012). Role of lipotoxicity in endothelial dysfunction.. Heart Fail. Clin..

[23] Kae-Woei Liang, Wayne H-H Sheu, Wen-Jane Lee, Wen-Lieng Lee, Chia-Po Fu, Jun-Sing Wang (2017). Differential expression of circulating vascular cell adhesion molecule-1 in subjects with coronary artery disease and cardiac syndrome X without known diabetes mellitus.. Biomarkers.

[24] Fotis Lampros, Agrogiannis Georgios, Vlachos Ioannis S., Pantopoulou Alkistis, Margoni Angeliki, Kostaki Maria, Verikokos Christos, Tzivras Dimitrios (2012). Intercellular Adhesion Molecule (ICAM)-1 and Vascular Cell Adhesion Molecule (VCAM)-1 at the Early Stages of Atherosclerosis in a Rat Model.. In vivo.

[25] Wang S-X., Tan L., Wang J., Zhong J-Q. (2016). Effect of levocarnitine on TIMP-1, ICAM-1 expression of rats with coronary heart disease and its myocardial protection effect.. Asian Pac. J. Trop. Med..

